# The roles of tissue resident macrophages in health and cancer

**DOI:** 10.1186/s40164-023-00469-0

**Published:** 2024-01-16

**Authors:** Minmin Cao, Zihao Wang, Wanying Lan, Binghua Xiang, Wenjun Liao, Jie Zhou, Xiaomeng Liu, Yiling Wang, Shichuan Zhang, Shun Lu, Jinyi Lang, Yue Zhao

**Affiliations:** 1 School of Basic Medical Sciences, Chengdu University of Traditional Chinese Medicine, Chengdu, China; 2 Department of Radiation Oncology, Radiation Oncology Key Laboratory of Sichuan Province, Sichuan Clinical Research Center for Cancer, Sichuan Cancer Hospital & Institute, Sichuan Cancer Center, Affiliated Cancer Hospital of University of Electronic Science and Technology of China, Chengdu, China; 3 School of Medicine, University of Electronic Science and Technology of China, Chengdu, China; 4 Guixi Community Health Center of the Chengdu High-Tech Zone, Chengdu, China

**Keywords:** Tissue resident macrophages, Bone-marrow derived macrophages, Monocytes, Homeostasis, Cancer

## Abstract

As integral components of the immune microenvironment, tissue resident macrophages (TRMs) represent a self-renewing and long-lived cell population that plays crucial roles in maintaining homeostasis, promoting tissue remodeling after damage, defending against inflammation and even orchestrating cancer progression. However, the exact functions and roles of TRMs in cancer are not yet well understood. TRMs exhibit either pro-tumorigenic or anti-tumorigenic effects by engaging in phagocytosis and secreting diverse cytokines, chemokines, and growth factors to modulate the adaptive immune system. The life-span, turnover kinetics and monocyte replenishment of TRMs vary among different organs, adding to the complexity and controversial findings in TRMs studies. Considering the complexity of tissue associated macrophage origin, macrophages targeting strategy of each ontogeny should be carefully evaluated. Consequently, acquiring a comprehensive understanding of TRMs' origin, function, homeostasis, characteristics, and their roles in cancer for each specific organ holds significant research value. In this review, we aim to provide an outline of homeostasis and characteristics of resident macrophages in the lung, liver, brain, skin and intestinal, as well as their roles in modulating primary and metastatic cancer, which may inform and serve the future design of targeted therapies.

## Introduction

Macrophages played pivotal roles in maintaining homeostasis through sensing various signals, phagocytosis and orchestrating subsequent immune response, accounting for only 1–5% in organs under steady state and the number can be as high as to 40% in cancer tissues [1–3]. The traditional view holds that tumor associated macrophages (TAMs) mainly arise from the differentiation of hematopoietic precursors [4, 5]. In the past decade, TAMs are considered to be derived from different origins with distinct phenotypes and divergent functionality, that embryonic-derived macrophages also contribute to the generation of TAMs in the pancreas, brain, lung and breast [5–8]. It is now accepted that TAMs can be either originated from long lived yolk-sac or fetal liver progenitors during organogenesis or recruited from bone marrow myeloid progenitors, namely tissue resident macrophages (TRMs) and bone marrow derived macrophages (BMDMs) respectively [1, 9–11]. Under steady-state conditions, resident macrophages, including brain microglia, liver Kupffer cells and epidermal Langerhans cells are locally self-maintained, independently of BMDMs across most organs in adult mice [12]. While TRMs of peritoneal and pancreatic stroma are both self-renewed and slightly replaced by BMDMs [1]. Intestinal resident macrophages are quite different in that they are constantly replaced by BMDMs [13].

Despite from their ontogeny differences, tissue resident macrophages varied from BMDMs in several aspects. Tissue resident macrophages are located at specific sub-tissular niches in each organ [1]. Alveolar macrophages are conjunct to alveolar epithelial cells, receiving GM-CSF from epithelial cells and clearing surface surfactants [14, 15]. Lymphatic vessel endothelial hyaluronan receptor (LYVE^+^) vascular associated macrophages and MHCII^+^ nerve associated macrophages has been identified due to their specified microanatomical niche across different organs [16]. Besides, unique tissue resident macrophages localization endowed with tissue specific functions that may not be easily replaced by bone marrow derived macrophages. For example, microglia are capable of mechanically prune neurons and remodel synaptic connectivity [17, 18]. Kupffer cells are equipped with machinery for uptake and recycling erythrocytes while recruited monocyte do not [19]. Osteoclasts are required for bone morphogenesis and hematopoietic niche maintenance [20]. Moreover, tissue resident macrophages are early witness and encounters during oncogenesis and micro metastasis formation, sensing cancer-related soluble products, while BMDMs can be recruited at relatively later stage of cancer progression [1, 3]. In a single-cell sequencing study, macrophages accumulated in cancer tissues demonstrate enhanced monocyte-like characteristics, apart from liver tumors, where the increased macrophage populations correspond to monocyte-derived macrophages, highlighting a cancer-type specific recruitment manner [21]. The time sequence of macrophages in tumor microenvironment from varied ontogeny may modulate tumor progression in different ways.

In this review, we outlined our current understanding for the roles of tissue resident macrophages of lung, liver, brain, skin and intestine in cancer. These five organs were selected for the extensive and in-depth cancer related research on particular tissue resident macrophages. Besides, the prevalence and mortality rate of malignancies in these five organs are among the top of public health concern worldwide.

## Pulmonary tissue resident macrophages

### Homeostasis of tissue resident macrophages in lung

Pulmonary macrophages play a crucial role in defending against pathogens and maintaining cellular debris clearance, ensuring lung homeostasis [22, 23]. The lung harbors three populations of tissue resident macrophages, namely alveolar macrophages (AMs), and interstitial macrophages (IMs) including vascular-associated interstitial macrophages, and nerve-associated interstitial macrophages (NAMs) [22, 24]. Among these, AMs represent the predominant population, accounting for 90–95% of pulmonary resident immune cells, and are mainly distributed in the lung's diffusing area [14, 25, 26]. Vascular-associated interstitial macrophages reside preferentially alongside blood vessels, while NAMs are located in proximity to nerves within the bronchial tree [22, 24]. Recent studies have indicated that both mouse and human AMs originate from embryonic precursors and occupy the alveolar niche before birth. Throughout adulthood, they undergo minimal proliferation in the absence of inflammation, relying on self-renewal mechanisms, but can be replenished by BM-derived cells under stressful conditions [11, 27–31]. On the other hand, IMs are believed to originate from the yolk sac and are subsequently replaced by fetal liver monocytes [32]. Depending on the specific subtype, IMs can either self-replenish without input from circulating monocytes or gradually undergo replacement by monocytes at varying rates and extents [33, 34]. Notably, a study conducted by Ural et al. during pregnancy demonstrated that NAMs are derived from the yolk sac during pregnancy and persist at least several months after birth [22]. Interestingly, NAMs in other organs, such as the skin and gut, exhibit self-renewing capacities and share common features with microglia [13, 35, 36].

### Characteristics of pulmonary resident macrophages

**Fig. 1:**
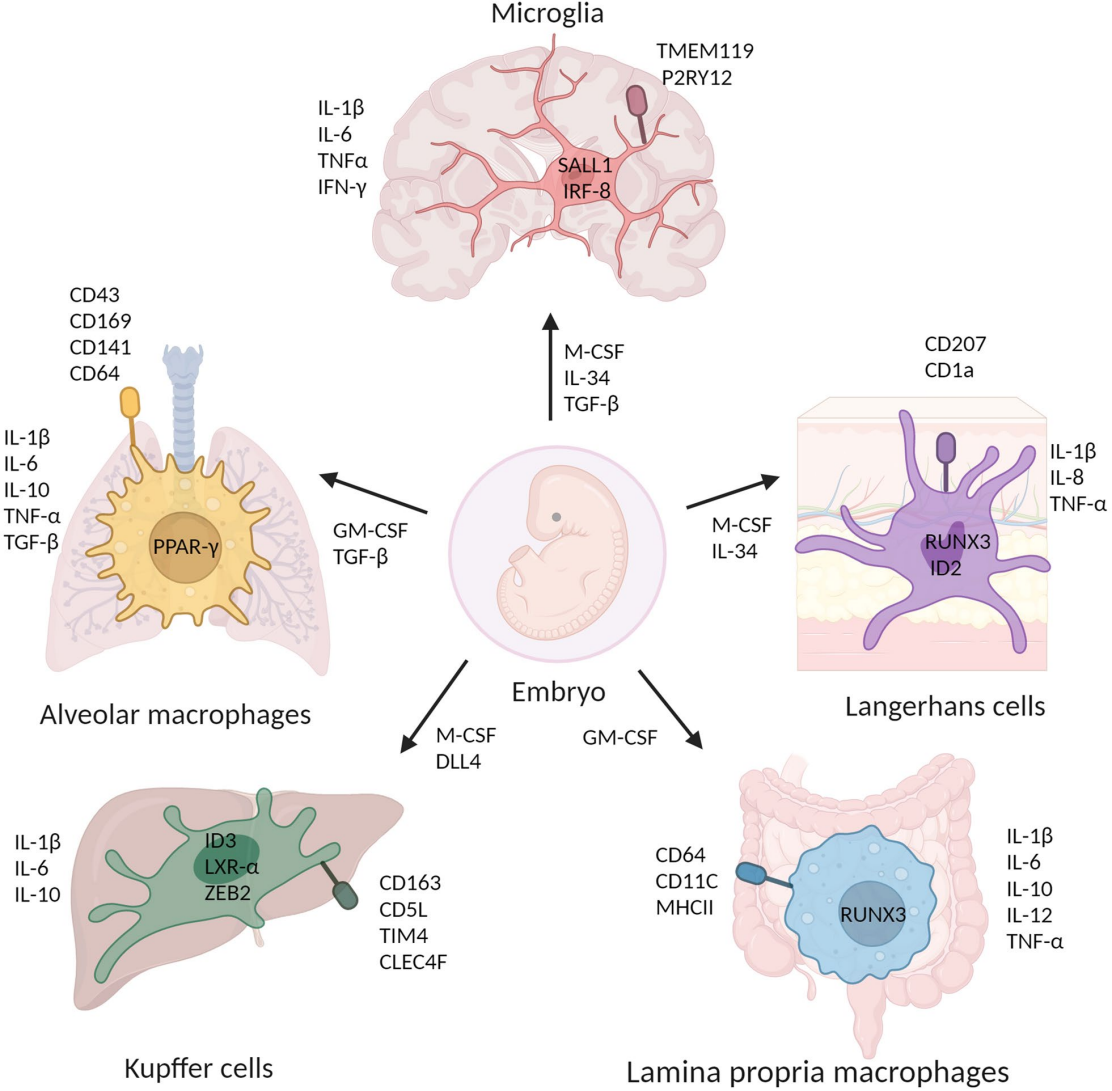
The identity of TRMs is determined by their specific niche of residence. TRMs in different tissues originate from the embryo and rely on distinct niche signals and transcription factors to facilitate their differentiation, survival and self-maintenance. Additionally, TRMs in the lung, liver, brain, skin, and intestine secrete various cytokines and exhibit distinct markers. These factors collectively define the specific identity of TRMs

Alveolar macrophages (AMs) rely on the activation of GM-CSF and TGF-βR signaling pathways, which in turn activate the transcription factor PPAR-γ, to facilitate their differentiation and survival [37]. Deficiencies in GM-CSF, either due to autoantibodies or gene deletions, can lead to the excessive accumulation of surfactant lipids and proteins produced by type 2 alveolar epithelial cells in the alveolar space, resulting in a condition known as pulmonary alveolar proteinosis [38–40]. Additionally, conditional deletions of TGF-βR at various time points in murine models have been found to halt the development and differentiation of AMs [41]. In contrast, the development and survival of IMs rely on the CSF1R signaling axis [22] (Fig. 1).

Different types of pulmonary tissue-specific macrophages exhibit distinct phenotypic and transcriptional features. AMs reside on the luminal side of the alveolar niche [32]. In humans, AMs are often characterized by the co-expression of HLA-DR, CD43, CD169, CD206, CD11b, CD141, CD64, and low levels of CD14, while in mice, they are identified by CD11C, CD64, F4/80, CD45, CD36, CD206, MERTK, and SIGLECF [32, 42]. The pan-cancer analysis of single myeloid cells across 15 human cancer types unveiled significant findings, indicating that AMs express genes such as FABP4, MARCO, MRC1( also known as CD206), MSR1 and PPAR-γ, which are involved in self-replenishment through the secretion of (TGF-β [43–45]. In addition, M2 AMs secrete anti-inflammatory mediators, such as IL-10, PGE2, and TGF-β, while M1 AMs produce inflammatory mediators, such as INOS, IL-1β, IL-6, TNF-α, and MIP1α [46]. Vascular-associated interstitial macrophages are recognized as CD206^+^LYVE-1^hi^MHCII^lo^CX3CR1^−^ cells, expressing an immunoregulatory signature, including TGF-β2, PLAUR and FCNA, while nerve-associated macrophages are characterized as CD206^−^LYVE-1^lo^MHCII^hi^CX3CR1^+^ cells, closely associated with nerve bundles or endings and highly express pro-inflammatory molecules such as IL-1β and CXCL12 with antigen presenting function. Nerve-associated macrophages secrete immunomodulatory IL-10 [22, 24, 37].

### Lung resident macrophages in cancer

Like monocyte derived macrophages, tissue resident macrophages such as AMs also possess substantial diversity in response to tumor-microenvironmental cues and employ considerable plasticity in orchestrating immune responses to cancer. Besides, a recent cross-tissue single-cell sequencing study indicated that LYVE1^+^ macrophages that are often HES1^+^, with overlapped features of fetal liver macrophages, highlighting the possibility that tissue resident macrophages can be reverted to an embryonic state with potential diversity and plasticity applicable to different functions [21]. Recent studies involving treatment non-small cell lung cancer (NSCLC) patients and a mouse model using KrasG12D and P53 null (KP) epithelial cells have highlighted the significant role of tissue-resident AMs in driving lung tumorigenesis [47]. In line with these findings, a subset of P16 and CXCR1-expressing AMs with features resembling cellular senescence have been identified as suppressors of the antitumor cytotoxic T cell response during Kras-driven murine lung cancer development [48]. Importantly, the removal of these senescent AMs has been shown to substantially diminish tumorigenesis. Furthermore, similar senescent AMs have been detected in early-stage treatment human NSCLCs. Lineage tracing experiments have revealed that as NSCLC lesions develop in mice, tumor-associated macrophages accumulate near tumor cells, facilitating invasion, epithelial-mesenchymal transition, and increased dispersion of tumor cells over time. Moreover, these tumor-associated macrophages induce potent regulatory T cell responses that confer protection to tumor cells against adaptive immunity [8, 47, 49]. AMs may promote lung tumors through ROS/BACH1/PDLIM2/STAT3 signaling pathway with impaired phagocytosis [50]. In a murine lung cancer model, the expression of Inhibin beta A (INHBA) is upregulated in AMs during tumor-bearing conditions, resulting in the secretion of activin A. Interestingly, this activin A secretion has been found to inhibit the proliferation of lung cancer cells. Additionally, experimental models have shown that the postnatal deletion of INHBA/activin A can restrict tumor growth [51]. AMs-derived extracellular vesicles containing SOCS3 inhibited STAT3 activation as well as proliferation and survival of lung adenocarcinoma cells. Recombinant SOCS3 delivered in synthetic liposomes inhibits in vitro proliferation and survival of lung adenocarcinoma cells and suppresses the malignant transformation of normal endothelial cells, suggesting its potential as a lung cancer treatment strategy [52]. Consistent with this notion, it has been observed that BMDMs facilitated metastatic tumor spreading in the lung cancer models, whereas TRMs supported proliferation of cancer cell at the primary tumor site [8]. Other single-cell sequencing studies have also identified TRMs-TAMs as macrophages resembling normal TRMs but with high expression of embryonic precursor features [47, 53], and their application in lung cancer has shown elevated expression of MARCO, scavenger receptors, and FABP4, similar to AMs [54, 55]. TRMs-TAMs promote tumor invasiveness by inducing Epithelial-mesenchymal transition (EMT) and recruiting regulatory T cells in the KP lung cancer model [56].

**Fig. 2:**
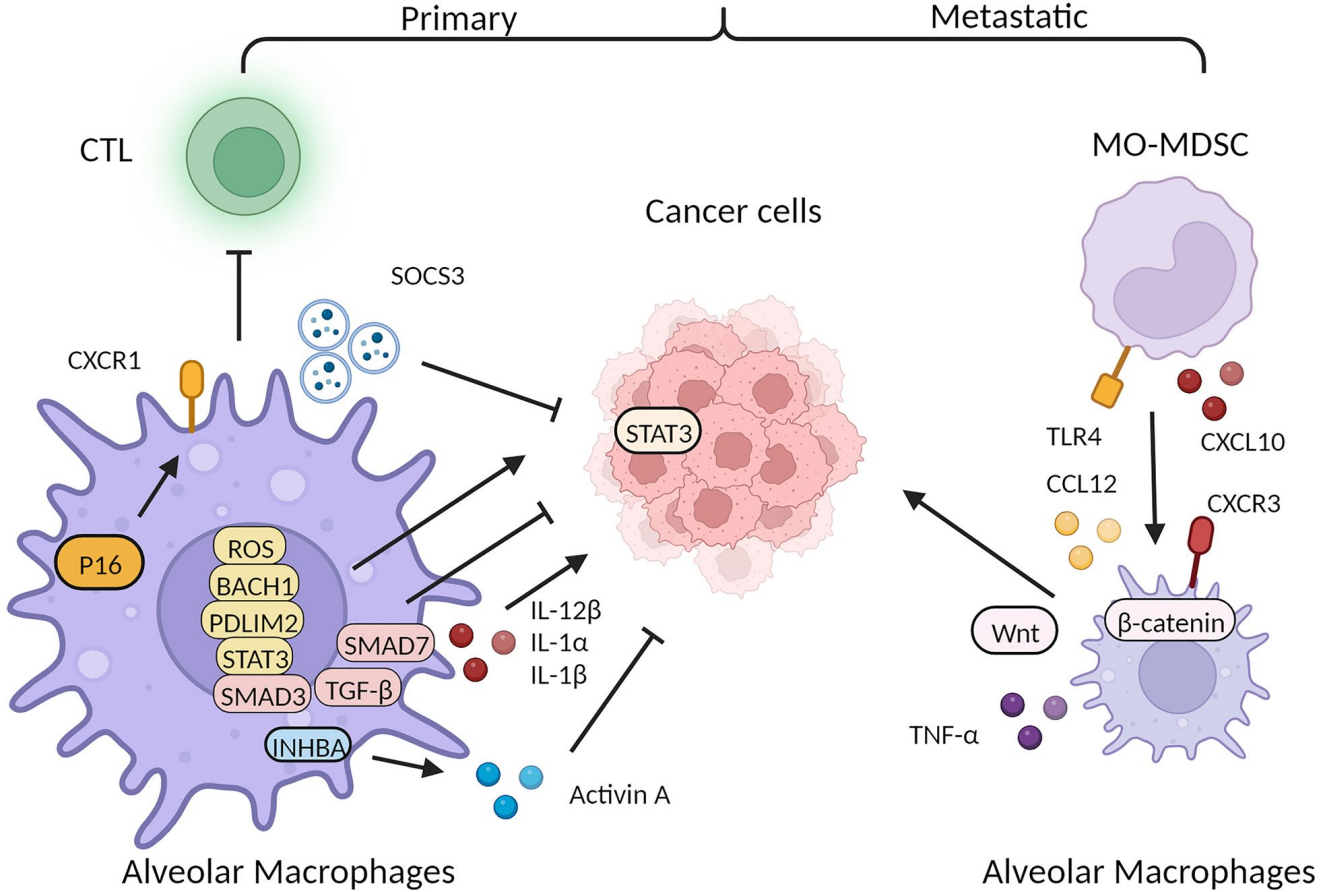
Dynamic crosstalk between pro- and antitumorigenic alveolar macrophages (AMs) and tumor cells in primary and metastatic cancers. In primary tumors, AMs expressing P16 and CXCR1 suppress CTL response and promoting the progression of lung tumor via the ROS, BACH1, PDLIM2 and STAT3 signaling pathway; Upregulation of INHBA expression in AMs leads to the secretion of activin A, which inhibits the proliferation of lung cancer cells. Additionally, AMs-derived extracellular vesicles containing SOCS3 suppresses STAT3 activation in cancer cells leading to inhibits the proliferation and survival of lung adenocarcinoma cells. Upregulation of pro-inflammatory genes, such as IL-12β, IL-1α, and IL-1β, and also anti-inflammatory genes, including Smad3 of the TGF-β signaling pathway, and downregulation of Smad7. During cancer metastasis, AMs are recruited from MO-MDSCs through the CXCL10-CXCR3 and TLR4-CCL12 axis, and contributing to the metastatic progression through the Wnt/β-catenin/TNF-α axis

In addition to primary pulmonary carcinoma, lung TRMs can play a crucial role in promoting metastatic lesions in the lungs. In a murine melanoma lung metastasis model, researchers have observed that resident AMs maintain the expression of a mixed pro-inflammatory/anti-tumor gene profile over time as the lesion grows, including upregulation of pro-inflammatory genes, such as IL-12β, IL-1α, and IL-1β, and also anti-inflammatory genes, including Smad3 of the TGF-β signaling pathway, and a considerable downregulation of the inhibitory Smad, Smad7 from the early to the late stage [57]. Conversely, IMs undergo a shift in their gene expression from an initial pro-inflammatory state, characterized by upregulation of genes such as SOCS1 and downregulation of CD38, IGF1, andCD206, to a later tumor-promoting profile, with substantial induction of Arginase-1. During metastatic growth, AMs within the macrophage pool gradually get replaced by infiltrative macrophages, which may contribute to metastatic progression [57]. Mechanistically, the WNT/β-catenin/TNF-α pro-metastatic axis in AMs has been implicated, revealing potential therapeutic implications for targeting tumors that are resistant to the anti-neoplastic effects of TNF-α [58]. Moreover, targeting the recruitment of Monocytic-myeloid-derived suppressor cells (MO-MDSC) through CXCL10/CXCR3 and TLR4/CCL12 axis in AMs holds promise as a therapeutic strategy to suppress lung metastasis [59] (Fig. 2, Table 1).

**Table 1:** Signaling pathway and function of tissue resident macrophages in cancer

Cell	Signaling pathway	Key function	Refs.
Aveolar macrophage	P16/CXCR1	Suppress the cytotoxic T cell response to pro-tumor	[48]
	ROS/BACH1/PDLIM2/STAT3	Promote tumor growth	[50]
	INHBA/activin A	Anti-tumor effects	[52]
	SOCS3/STAT3	Inhibition of proliferation and survival of cancer cells	[51]
	Upregulation IL-12β, IL-1α, and IL-1β	Pro-inflammatory in metastasis	[57]
	Upregulation of Smad3, TGF-β and downregulation of Smad7	Anti-inflammatory in metastasis	[57]
	WNT/β-catenin/TNF-α	Pro-metastatic environment	[58]
	CXCL10/CXCR3 and TLR4/CCL12	Suppresses lung metastasis	[59]
Kupffer cell	FOLR2^+^ macrophage express CXCL12, CXCL16, and CD86	Engage in immunosuppressive interactions with Tregs	[93]
	PD-L1/PD-1 and TIM-3/Galectin-9	Inhibit the antitumor response	[94–96]
	TREM-1 deficiency reduced IL-1β, IL-6, CCL2, and CXCL10	Contribute to anti-tumor response	[97]
	ROS and TNF	Cause intrahepatic cholangiocarcinoma	[73]
	Early-stage KCs depletion modulated INOS and VEGF	Enhance metastasis progression	[99]
	Late-stage KCs depletion increased CD3 cells infiltration	Impede metastasis growth	[99]
	Mafb- and Maf- dual knockout	Exhibit anti-tumor response by reverse KCs function	[101]
Microglia	EGF and STI1	Promote glioblastoma cell invasion	[143, 144]
	TGF-β	Promote glioma cells migration	[145]
	CCL2/CCR2/IL-6	Promote glioma invasion	[147, 148]
	IL-6/JAK2/STAT3	Promote a pro-metastatic environment	[154]
	LPS-activated	Induce apoptosis in metastatic lung cancer cells	[153]
	ANXA1/STAT3	Enhance metastatic cell migration	[155]
	TGF-β	Fosters tolerance towards metastatic melanoma cells	[156]
	CPG-C	Combats brain metastases	[157]
Langerhans	CD4^+^ and CD8^+^ T cells enhance production of IFN-γ	Anti-tumor immune response	[199, 200]
	TLR3 ligand polyinosine	Enhance the CD8^+^ T cell response	[201]
	PAK1	Alter LCs functions in skin carcinogenesis	[206]
	CD207/liposomes 22 vaccine	Induce protective immune responses against tumors	[208]
Gut resident macrophage	MR, CD163/CCL17, CCL22, CCL24, IL-10 and TGF-β	Promote tumor development	[230]
	C-MYC/VEGF, HIF-1α, and TGF-β	Regulates the expression of pro-tumoral genes	[231]
	IL-34 and IL-10	Enhance tumor proliferation	[236]
	CCR2-independent F4/80^+^MHCII^lo^ subset	Promote tumor progression	[237]
	ERK/S100A8/A9	Enhance tumor migration	[240]
	JAK2/STAT3/FOXQ1	Promote its migration and invasion	[241]

## Liver tissue resident macrophages

### Homeostasis of tissue resident macrophages in liver

With the largest population of resident tissue macrophages in all solid organs, liver-resident macrophages perform various functions crucial for liver homeostasis, including the clearance of erythrocytes and debris, regulation of iron and lipid metabolism, maintenance of immune tolerance, and sensing tissue damage [60–63]. KCs are primarily of embryonic origin and self-maintain throughout life [64–66], reside within the lumen of the liver sinusoids and do not originate from circulating monocytes under steady state [67–69]. While they share common functions with tissue macrophages, such as responding to tissue damage and antigen presentation, KCs also have specialized roles in iron scavenging and uptake of digested particles from the portal blood [63]. Monocyte-derived macrophages, on the other hand, are mainly localized at the portal triad in the healthy liver and also contribute to iron and cholesterol metabolism [70, 71]. Kupffer cells are major producers of cytokines such as IL-1β, IL-6, IL-10 and TGF-β1, chemokines such as CXCL1, CXCL2, CXCL8, CXCL16, CCL2 and other bioactive molecules including YAP in response to appropriate stimulation, playing a crucial role in maintaining liver homeostasis [72–75]. KCs can be replaced by monocyte derived KCs in disease model, for example, only when the proliferation of embryo-derived KCs is impaired, KCs secrete chemokines like CCL2, which leads to the excessive infiltration of LY6C^hi^ bone marrow monocytes and subsequent liver injury [76–78]. It is noteworthy that repopulation of monocytes appears to be relatively inefficient compared to proliferation of resident cells and they lack the necessary machinery for the uptake and recycling of erythrocytes [68, 79, 80].

### Characteristics of liver resident macrophages

Kupffer cells depend on the activation of the macrophage colony-stimulating factor 1 receptor, which is stimulated by M-CSF and IL-34, as well as rely on the transcription factor ID3 and LXR-α to facilitate their differentiation, survival and self-maintenance [10, 37]. Inactivation of ID3 impairs the development of liver macrophages and leads to selective KCs deficiency in adults [31]. Additionally, the transcription factor ZEB2 maintains the expression of LXR-α, its loss results in change in KCs identity and their disappearance [81]. Recent study has shown that the induction and maintenance of KCs identity require a combination of interactions involving DLL4, TGF-β family ligands, and endogenous LXR ligands [19]. Moreover, the expression of DLL4 by liver sinusoidal endothelial cells is crucial for driving the differentiation of monocytes into KCs, as well as this process is facilitated by the rapid induction of LXR-α expression and an increased responsiveness to LXR-α-inducing signals produced by hepatic stellate cells [19, 79, 81].

Kupffer cells exhibit specific surface markers and transcriptional features that differ from other TRMs. The transcription factor CLEC4F has been identified as a specific marker for KCs, while TIM4 is expressed in various long-lived tissue-resident macrophage subsets, throughout development and postnatally [81–84]. In mouse, KCs are characterized by F4/80^+^CD11B^+^CD68^+^TIM4^+^CLEC4F^+^ surface phenotype [72, 85], as well as exhibit a diverse repertoire of immune receptors, including toll-like receptors (TLRs) like TLR4 and TLR9, scavenger receptors and complement receptors, which play a vital role in maintaining liver tolerance by orchestrating the induction of regulatory T cells [61, 86]. In contrast, the heterogeneity of human liver macrophages is less well-defined compared to mouse Kupffer cells. In humans, KCs have been defined as a CD163^+^MARCO^+^CD5L^+^TIM4^+^CLEC4F^+^ macrophage population in human liver based on single-cell sequencing studies conducted by three separate research groups [87–89]. In addition, to demonstrate the heterogeneity of KCs, the relevant study revealed the existence of two distinct KC populations in the steady-state murine liver: KC1 (CD206^lo^ESAM^−^), and KC2 (CD36^hi^CD206^hi^ESAM^+^). Notably, the CD36^hi^CD206^hi^ESAM^+^ KC2 subset is specifically involved in the regulation of liver fatty acid metabolism [90]. However, these markers may not be sufficient to distinguish KCs from recruited macrophages, highlighting the need for further research in this area.

### Kupffer cells in cancer

Numerous studies have proven that significant role of Kupffer cells (KCs) in the development of Hepatocellular carcinoma (HCC). The latest research has shown that KCs, acting as incomplete APCs, can induce CD8^+^ T cell tolerance and revert T cell exhaustion in the context of HBV infection model [91, 92]. In addition, two clusters of FOLR2^+^ TAM1 populations with different origins were identified: monocyte derived while another appears to be embryonic-origin tissue-resident macrophages. FOLR2^+^ TAM1 displayed immunosuppressive interactions with Tregs, supporting the onco-fetal reprogramming of tumor microenvironment (TME) in HCC [93]. It can be said unequivocally that KCs inhibit the antitumor response by activating signaling pathways involving PD-L1/PD-1 and TIM-3/Galectin-9 in T cells [94–96]. In response to stimulation from cancer cells, the expression of the TREM-1 increases, promoting KCs activation and HCC progression, while TREM-1 deficiency has been found to decrease the release of IL-1β, IL-6, CCL2, and CXCL10 by KCs, resulting in suppressed HCC growth [97]. Others studies suggest that ROS and paracrine TNF produced by KCs contribute to biliary tract cell proliferation and may eventually causing intrahepatic cholangiocarcinoma [73].Overall, KCs play a vital role in the liver immune microenvironment by secreting an array of soluble proteins such as cytokines, chemokines, and growth factors.

**Fig. 3:**
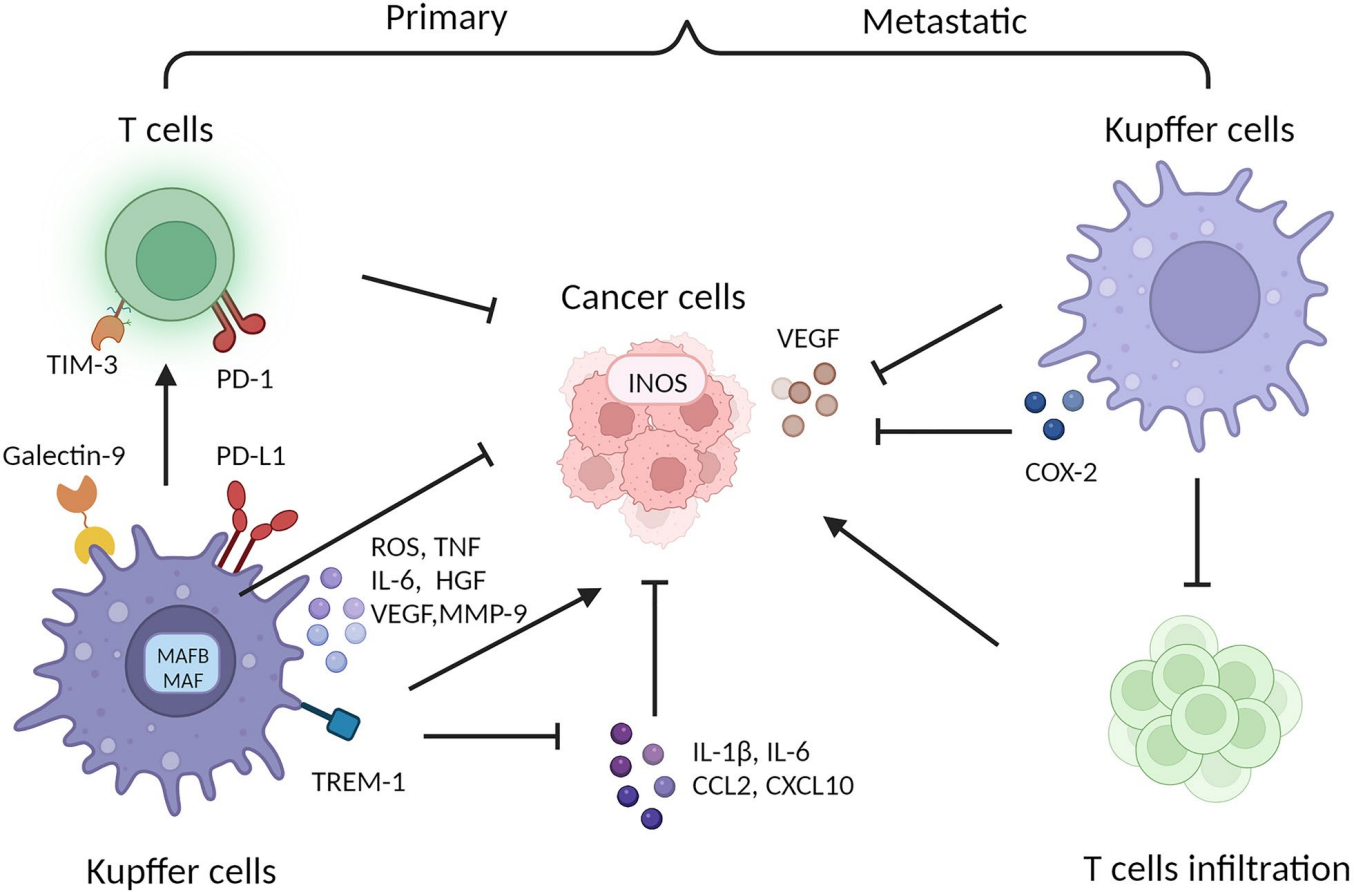
Dynamic interaction between pro- and antitumorigenic Kupffer cells (KCs) and tumor cells in primary and metastatic cancer. In primary tumors, KCs inhibit the anti-tumor response by activating signaling pathways involving PD-L1/PD-1 and galectin-9/TIM-3 in T cells. Stimulation from cancer cells leads to an increase in TREM-1 expression in KCs, promoting the progression of hepatocellular carcinoma (HCC). TREM-1 deficiency results in the reduced release of IL-1β, IL-6, CCL2, and CXCL10 by KCs, thereby suppressing HCC growth. ROS and TNF produced by KCs contribute to tumor cell proliferation. Constructed MAFB- and MAF-targeting dual sgRNA CRISPR/CasΦ vector in KCs achieved therapeutic effects. During cancer metastasis, KCs was found to decrease tumor growth by altering the expression of INOS and VEGF in cancer cells. Conversely, KCs increased tumor growth through decreased infiltration of T cells and upregulation of COX-2 expression

Apart from HCC, KCs also play a significant role in liver metastasis of various cancers. In the gastric cancer liver metastasis, KCs have been suggested to undergo a transition between M1 polarization, characterized by adherence, tumor cell phagocytosis, and induction of cell apoptosis, and M2 polarization, characterized by secretion of IL-6, HGF, VEGF, and MMP-9. This transition allows KCs to exert bidirectional effects on tumor cells through different mechanisms [98]. In an orthotopic murine model of colorectal cancer liver metastasis, a study investigating KCs depletion revealed the dualistic nature of KCs in influencing tumor growth. The early depletion of KCs was observed to enhance tumor progression by modulating the expression of INOS and VEGF within the TME. Conversely, late-stage KCs depletion exhibited a contrasting effect, impeding tumor growth by augmenting the infiltration of CD3 infiltrating immune cells and promoting apoptosis [99]. Interestingly, in a WAG/Rij rat syngeneic model of CRC liver metastasis induced by staged hepatectomy, it was observed that KCs expressed COX-2, while BMDMs primarily expressed Arginase-1 [100]. By utilizing the CRISPR/CasΦ mechanism to edit tumor-associated genes in KCs, it was observed that specific clearance of KCs plays an important anti-tumor role in the early stage of liver metastasis, while dysfunction occurs in the later stage of liver metastasis. In this research, the authors overcome KC dysfunction and elicit remarkable curative effects against several types of metastatic liver cancer in mice by using constructed MAFB- and MAF-targeting dual sgRNA CRISPR/CasΦ Vector [101]. Therefore, finding proper ways to regulate KCs in anti-cancer treatment will pose a major challenge (Fig. 3).

## Central nervous system (CNS) resident macrophages

### Homeostasis of tissue resident microglia in CNS

As part of the CNS’s innate immune system, microglia are vital for preserving immune defense and resolution, neuronal support, tissue maintenance, and synaptic integrity [102–105]. Microglia, which are widely distributed throughout the brain parenchyma, comprising approximately 10–15% of all glial cells and are commonly recognized as the CNS’s tissue-resident macrophages [106]. Contrary to studies conducted in healthy adult mice, research on human microglia has revealed significant spatial heterogeneity in their transcriptional programs, particularly in relation to grey and white matter regions [107–110]. Consistent with other TRMs origin, microglia colonize the brain as embryonic microglia during the initial phases of development [106, 111]. These immature myeloid cells, characterized by their high proliferative potential and amoeboid morphology, migrate into the brain through various routes. In mice, they enter via the meninges and ventricles [112–114], Similarly, in humans, microglia traverse through the leptomeninges, choroid plexus, and ventricular zone [115–117]. The initial colonization of microglia primarily takes place in the white matter regions, including the internal capsule, external capsule, and cerebral peduncle. As microglial cells undergo proliferation and migration, they subsequently extend their colonization to the subplate and cortical plate regions in both radial and tangential manners [117]. Microglia release trophic factors that promote neuronal circuit formation and survival, including IGF-1, which supports the postnatal survival of layer V cortical neurons [113]. Microglia possess the ability to detect harmful stimuli and mount a response by producing inflammatory cytokines such as TNF-α, IL-6, IL-1β, IFN-γ, and several chemokines such as CCL2, CX3CL1, CXCL10 [118]. Additionally, microglia, acting as accessory cells, can induce programmed cell death and clear cellular debris. This has been observed in the developing chick eye, where microglia release nerve growth factor to trigger apoptosis of retinal nerve cells [119], and in the murine hippocampus, where microglia induce programmed cell death in neurons [120]. Microglia actively participate in neuronal pruning during development, respond to synaptic activity and plasticity, and play a crucial role in maintaining synaptic homeostasis. They achieve this by releasing trophic factors and synaptogenic signals, including DAP12 signaling [121] 0.4.2 Characteristics of CNS resident microglia.

Microglia sustain their population by means of their extended lifespan and limited self-renewal capabilities within their microenvironment, primarily through the activation of CSF1R signaling pathways [106, 122–124]. The transcription factor SALL1 and IRF-8, expressed exclusively by microglia, is involved in regulating microglial identity and physiological properties, distinct from other members of the mononuclear phagocyte system or other CNS-resident cells in the CNS [125, 126]. Simultaneously, the transcription factors IRF-8 and PU.1 have an impact on CNS microglia by regulating their abundance, activation state, and developmental processes [111, 127]. It plays a vital role in maintaining microglial homeostasis and may control their activation through mechanisms involving apoptosis-related genes [111, 128]. Mouse models with IRF-8 deficiency (IRF-8 KO mice) demonstrate a significant loss of microglial signature genes accompanied by severely altered microglial morphology [129].

In the latest study, TMEM119 and Purinergic receptor P2RY12 are gaining ground as presumedly more specific microglia markers [130]. In adult mouse, microglia have been extensively characterized as TMEM119^+^P2RY12^+^CX3CR1^+^MRC1^−^ [108, 131], while TMEM119^+^P2RY12^+^CX3CR1^+^CD206^−^ in adult human [110, 122]. Furthermore, distinguishing microglia residing in the CNS from peripheral macrophages in tissues is challenging due to their shared expression of several markers, including CD11B, F4/80, CX3CR1, CD45, and Ionized calcium-binding adapter molecule 1 (IBA-1) [132]. However, studies have reported that CD44 is exclusively expressed by infiltrating cells and not by resident microglia [133]. Another study comparing the gene transcription profiles of adult microglia and peripheral cells revealed the absence of CD169 in microglia [134]. In certain situations, quantitative discrimination of markers is possible, such as detecting subtle differences in CD45 protein levels between CD11B^+^CD45^lo^ in microglia and CD11B^+^CD45^hi^ in macrophages from the adult brain [135, 136]. However, caution must be exercised as CD45 expression in microglia increases during inflammation and aging [137, 138]. Transcription factors PU.1 and MYB have also been identified as differentiating factors for microglia (PU.1-dependent transcription) and peripheral macrophages (MYB-dependent transcription) [133, 139, 140]. Therefore, discerning the unique characteristics of microglia in comparison to other myeloid cells is of utmost importance for comprehending brain development and diseases.

### CNS resident microglia in cancer

Microglia, as the largest population of TAMs in the CNS, have been recognized for their role in promoting tumor proliferation and invasion in the initiation and progression of brain tumors [141, 142]. In an vitro study, it was reported that murine glioma cells moved threefold more easily when exposed to microglial and migrated faster with a higher rate compared with tumor cells incubated without microglia [142]. Further investigations, have revealed that microglia can modulate tumor growth by secreting various cytokines. For instance, microglia were found to stimulate glioblastoma cell invasion by releasing EGF [143]. STI1, a ligand of the cellular prion protein, is synthesized and secreted by microglia to enhance the proliferation and migration of glioblastomas both in vitro and in vivo [144]. TGF-β released by microglia could promote glioma cells migration through strengthening the expression and function of integrin in co-culture systems [145]. Additionally, microglia could also be converted into a pro-tumorigenic phenotype by CSF1 released by tumor cells [146]. Human glioma cells induce microglia to secrete and release IL-6 through CCL2/CCR2 axis, thus promoting glioma invasion [147, 148]. In a mouse glioblastoma model primarily containing TRMs, combined inhibition of MERTK with radiotherapy demonstrated a significantly pronounced growth delay compared to radiotherapy alone [149]. In addition, inhibiting BMDMs could delay the recurrence of glioblastoma after radiotherapy due to the increased abundance of BMDMs post-irradiation [150]. However, some BMDMs probably undergo transformation into TRMs during growth, implying a functional switch that poses challenges to TAMs-based targeted therapies [151].

**Fig. 4:**
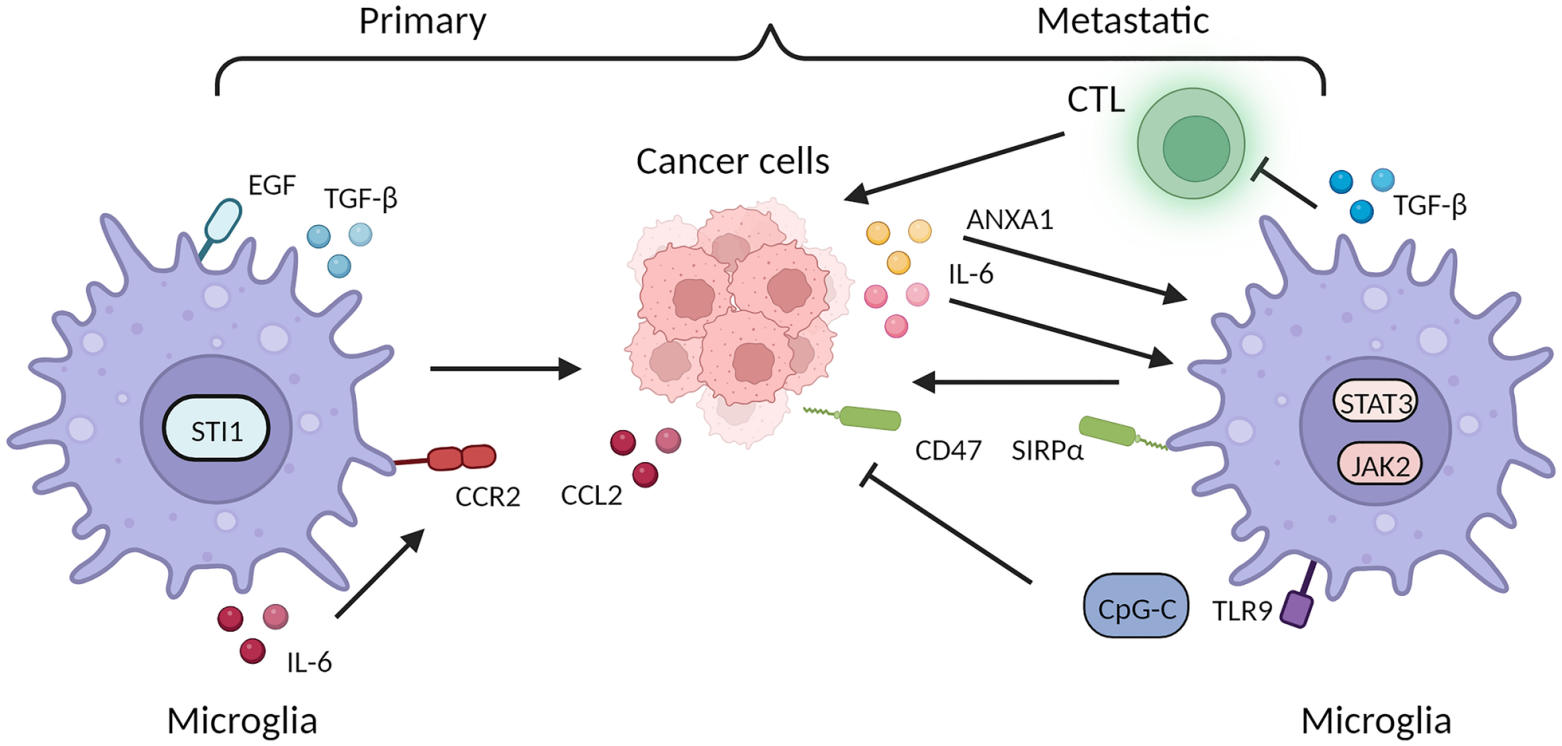
Dynamic interplay between pro- and antitumorigenic microglia and tumor cells in primary and metastatic cancers. In primary tumors, microglia enhance the proliferation and migration of cancer cells in both in vitro and in vivo settings by releasing TGF-β, EGF, and STI1. Cancer cells induce microglia to secrete and release IL-6 through the CCL2/CCR2 axis, thereby promoting glioma invasion。During cancer metastasis, tumor cells stimulate the JAK2/STAT3 signaling pathway in microglia by secreting IL-6 and promote tumor cell migration through the secretion of ANXA1, which reduces microglial migration and activates the STAT3. Elevated expression of TGF-β in microglia fosters tolerance towards cancer cells by anti-tumor CTL. CpG-C, inhibits brain metastasis by activating microglia, while the CD47-SIRPα axis acts as a crucial innate immune checkpoint suppressing phagocytic activity in myeloid cells

In addition to promoting tumor proliferation in primary intracranial tumors, microglia also exert a tumorigenic effect in brain metastasis. Various primary cancers, such as lung, melanoma, and breast cancer, can colonize the brain, with lung cancer showing the highest incidence of brain metastasis [152]. Microglia, when exposed to metastatic lung cancer in the brain, exhibit considerable activation and an increased number of cells labeled with the specific microglial marker [153]. Moreover, in the study of non-small cell lung cancer develop brain metastasis (NSCLC-BM), researchers found that brain-specific metastatic cells A549-F3 induce polarization of microglia towards an M2 phenotype characterized by high expression of CD206 and Arginase-1 via the IL-6/JAK2/STAT3 signaling pathway and microglia, in turn, promote NSCLC-BM development by affecting the colonization of metastatic cells [154]. On the contrary, the supernatant from LPS-activated microglia in vitro induces apoptosis in metastatic lung cancer cells in a dose- and time-dependent manner. Yet at lower concentrations, it demonstrates trophic effects, leading to some cancer cells becoming resistant to microglial cytotoxicity [153]. Microglia activation, also observed prior to brain metastasis in MMTV-Wnt1 mice models, is promoted by ANXA1 secreted from metastatic cancer cells, enhancing tumor cell migration. This process can be inhibited by silencing ANXA1 or using inhibitors, leading to reduced microglial migration and activation of STAT3 [155]. Concurrently, elevated expression of TGF-β in microglia fosters tolerance towards these metastatic melanoma cells by anti-tumor cytotoxic T cells [156]. Nonetheless, microglia can potentially be converted into tumor-unsupportive cells depending on the microenvironmental conditions and disease states. Recent research has shown that systemic administration of CPG-C, a toll-like receptor 9 agonist, combats brain metastases by activating microglia, which play a pivotal role in tumor suppression and phagocytosis upon direct tumor interaction. This is supported by elevated gene expression related to these processes both in vitro and in vivo [157]. Recent studies have highlighted the role of microglia as effector cells with a crucial role in anti-tumor responses. The CD47/SIRPα axis, a key innate immune checkpoint that suppresses phagocytic activity in myeloid cells [158]. Blocking this axis with anti-CD47 antibodies inhibits tumor growth and enhances survival in mice with human-derived glioblastoma multiforme cells by re-educating glioma-associated microglia [159]. Targeting the CD47/SIRPα axis shows promise in preventing various adult and pediatric brain tumors [160]. Of note, single-cell technology has unveiled that monocyte derived macrophages (MDMs) but not microglias play a crucial role in providing favorable signaling cues such as CD40 to Th cells and controlling the proliferation of metastatic cancer cells. Additionally, they contribute to the modulation of the TME in the brain by supporting the function of blood vessels, that perivascular M1-like MDMs exhibited higher CD40 expression than those further away from blood vessels, whereas perivascular M2-like MDMs expressed high levels of OX40L (also known as CD134L). Notably, a distinctive subset of macrophages expressing myeloperoxidase has been identified, which is associated with long-term survival [161]. The re-educating of microglia offers a more promising and positive therapeutic approach compared with conventional therapies that target the depletion of microglia to suppress their tumorigenesis properties. However, one of the major challenges in understanding the role of microglia in tumors is the lack of specific surface markers to distinguish microglia in the tumor microenvironment from bone marrow-derived macrophages [162]. The development of single-cell sequencing and transcriptome sequencing technology will help clarify the molecular mechanisms of cross-talk between microglia, the tumor microenvironment, and tumor cells. Due to their strong plasticity, microglia will likely become one of the most promising immunotherapeutic targets in the future (Fig. 4).

## Skin tissue resident macrophages

### Homeostasis of tissue resident macrophages in skin

Skin resident macrophages have diverse functions in maintaining skin homeostasis, inducing immunogenic, tolerant responses and involved in the development of skin diseases, such as psoriasis, atopic dermatitis, and psoriasis-like dermatitis [163–165]. They consist of two main cell types known as Langerhans cells (LCs) and dermal macrophages (DMs), serving as the frontline defense in the cutaneous immune system [37]. As the majority of skin tissue macrophages, LCs exhibit unique characteristics shared by both macrophages and DCs [166]. They are mainly located in the middle and upper part of the epidermis, residing between the epidermal spinous cells, and can also be found in the dermis, oral mucosa, vaginal epithelium and esophagus while DMs predominantly populate the dermis [36, 167]. Furthermore, LCs constitute approximately 3–5% of all nucleated cell in the adult epidermis [168]. LCs are thought to originate from hematopoietic precursors that colonize the skin during early embryonic development. The initial population of LCs is derived from yolk sac-derived CD16^+^ myeloid progenitor cells, which populate the skin prior to the onset of fetal liver hematopoiesis. During embryogenesis, these cells are largely replaced by CD14^+^ fetal hepatic mononuclear cells. Thus, the decline of adult LCs is mainly due to fetal hepatic mononuclear cells, with a smaller contribution from fetal yolk sac monocytes [169]. Lineage tracing studies have provided compelling evidence challenging the conventional notion that adult mouse LCs originate solely from bone marrow (BM)-derived DCs precursors. Instead, these studies have revealed that adult LCs are primarily derived from two distinct developmental sources: embryonic yolk sac-derived macrophages and fetal hepatic monocytes. This finding highlights the remarkable developmental plasticity of LCs and underscores the diverse origins contributing to their population in adult skin [170, 171]. However, recent breakthrough research has revealed that murine LCs present in mucosal epithelia are derived from circulating BM precursors and undergo continuous replenishment [172, 173]. Tongue LCs play a crucial role in antifungal immunity [174] and exhibit a protective role against cancer [175]. On the other hand, DMs primarily provide protection to newborn infants immediately at birth, safeguarding the outermost body surface from contact with bacteria colonizing the maternal genital tract and the skin [176]. Unlike LCs, DMs cannot migrate into the skin-draining lymph nodes and have limited antigen processing and presentation capabilities to T cells [177]. The single-cell transcriptomics, fate mapping, and imaging studies have revealed that DMs self-maintained with minimal postnatal input from hematopoietic stem cells but receive continuous contributions from circulating monocytes [36, 178]

### Characteristics of skin resident macrophages

The murine model has shown that the development and differentiation of Langerhans cells (LCs) are dependent on several transcription factors associated with TGF-β1 signaling, including PU.1, BMPR1A/ALK3, RUNX3, and ID2, as well as the interaction between CSF1 and IL-34 [179–182]. Some studies have demonstrated that PU.1 regulates LCs differentiation by controlling the expression of the critical TGF-β-responsive transcription factor, RUNX3 [183]. However, it has been found that the TGF-β1/Smad3 signaling pathway does not significantly affect LCs homeostasis and maturation. Further investigations have shown that blocking Smad2 or Smad4 alone, or in combination, in LCs lineages does not have a significant impact on the maintenance, maturation, antigen uptake, and migration of LCs in steady-state conditions, both in vivo and in vitro. However, disruption of the Smad2 and Smad4 pathways in the myeloid system leads to notable inhibition of BM-derived LCs in the inflammatory state [184]. Additionally, during epidermal ontogeny, the spatial and temporal availability of TGF-β family members, along with Notch ligands, collaborate to promote LCs differentiation [185, 186]. LCs are considered to be macrophage with DCs functions, as they originate from a MAFB-expressing progenitor, indicating a macrophage origin, as well as express the transcription factor ZBTB46, which reinforces their DCs identity [187, 188]. LCs are self-renewing in homeostatic conditions and are long-lived cells that can migrate and mature into DCs [189]. Dermal macrophages rely on the activation of the CSF1R and the cytokines on IL-10 and IL-4 for self-sustainment proliferation for self-maintenance [178], while LCs secreted IL-1β, low levels of TNF-α and IL-8, but not IL-6 or IL-10 [190].

Langerhans cells (LCs) possess distinct markers that aid in their identification. Specifically, the c-type lectin receptor langerin (CD207), which is a novel interferon-stimulated gene, serves as a specific marker for distinguishing LCs from other DCs subsets [191–193]. Moreover, LCs are characterized by the presence of unique rod- or tennis racket-shaped Birbeck granule in electron microscopy [194]. By employing single-cell sequencing and mass cytometry analysis on human LCs derived from CD34^+^ hemopoietic stem cells obtained from the cord blood, researchers have successfully identified four distinct subgroups of human LCs: LC1 (CD207^hi^, CD1a, EPCAM), LC2 (CD207^lo^, CD1c, CD1b, HLA-DR), activated LCs (Alc) (CCR7^lo^, CD83, CD40), and migratory LCs (migLC) (CCR7^hi^, CXCR4) [195]. LCs play a crucial role in inducing tolerance to protein antigens in intact skin through the action of Langerin. Additionally, targeting LCs via Langerin may hold potential for regulating systemic immune responses [196]. The LC1 and LC2 subgroups can be discerned by their distinct expression patterns of the C-type lectin receptor “Langerin,” the CD1 family of non-classical antigen-presenting receptors (CD1a, CD1b, CD1c), and EPCAM. The LC1 subset demonstrates elevated levels of langerin, CD1a, and EPCAM, whereas the LC2 subset exhibits lower langerin expression and higher levels of CD1c and CD1b [197].In addition, Early growth response 1 (EGR1) and Notch pathways have been found to have a significant impact on the bifurcation of LC1 and LC2, where LC1 may function as “effector LCs” with classical LCs functions, and LC2 may act as “regulatory LCs” that primarily play an immunoregulatory role under inflammatory conditions. In most cases, LC1 and LC2 subsets work collaboratively to interact with the external environment and orchestrate immune responses, thereby contributing to the maintenance of skin homeostasis [195]. In contrast to human LCs, mouse LCs lack CD205 and CD207 expression, and instead express CD14, CD204, and low levels of MHCII and TLR4 molecules at birth. Intriguingly y, the CD204^+^ and CD14^+^ LCs disappear after four days, and MHCII, CD11c, and CD207 are acquired during the first week of life [198].

### Langerhans cell in cancer

**Fig. 5:**
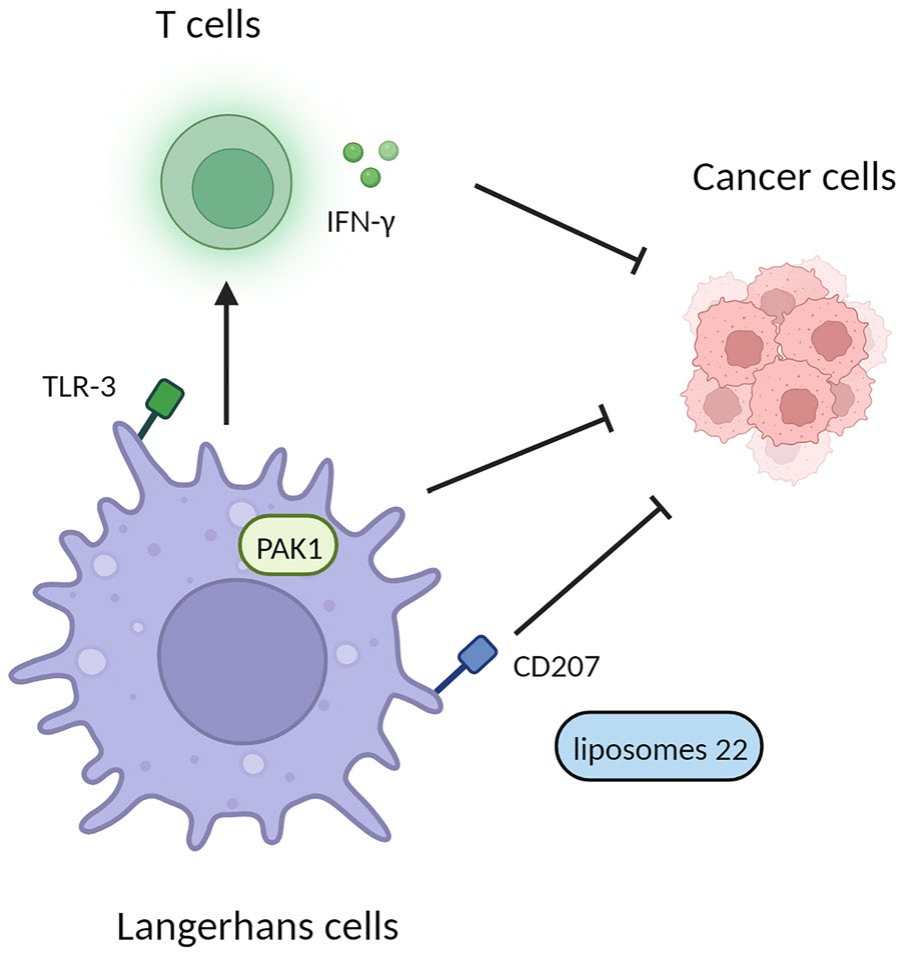
Langerhans cells (LCs) exhibit anti-tumor effects. LCs induce proliferation of T cells and enhance the production of IFN-γ. The potency of LCs in T cell response is further enhanced through activation with TLR-3. PAK1 expressed on LCs regulates the number of epidermal stem cells, thereby exerting inhibitory effects on tumor proliferation. CD207 and a glycomimetic Langerin ligand liposomes 22 can serve as transcutaneous cancer vaccine agents via inducing protective T cell immunity

The important role of skin tissue resident macrophages in skin cancer has been highlighted in a recent study on human Squamous cell carcinoma (SCC). The study findings revealed that LCs, a subset of DCs, can induce proliferation of CD4^+^ and CD8^+^ T cells and enhance production of IFN-γ more efficiently compared to other DCs subsets. LCs were discovered to possess a heightened capacity for inducing type 1 T cell responses within the SCC microenvironment, subsequently exerting a suppressive effect on antitumor activity [199, 200]. The potency of LCs to enhance the CD8^+^ T cell response can be further augmented by activating LCs with the toll-like receptor 3 ligand polyinosine [201]. Additionally, LCs have been found to promotes epithelial DNA damage and squamous cell carcinoma by metabolizing carcinogenic agents [202]. It is predicted that melanoma fail to activate the migration of LCs to lymph nodes until the tumor reaches a critical size, which is determined by a positive TNF-α feedback loop within the melanoma in silico model [203]. In non-melanoma skin cancer, a higher presence of LCs was observed in Basal cell carcinoma (BCC) cases compared to SCC. The reduction of Langerhans cells in SCC may indicate their role as a barrier against metastasis, whereas the marked reduction of LCs in SCC compared to BCC suggests their potential implicated in the intricate processes of non-melanoma skin cancer development and progression [204, 205]. P21-activated kinase 1 (PAK1), previously reported to have oncogenic activity in various types of cancer, was found to regulates the number of epidermal stem cell by altering LCs properties and functions in skin carcinogenesis. This underscores the emerging significance of PAK1 in the regulation of LCs, as well as its potential holds great promise for the treatment of skin immune diseases and the management of carcinogenesis [206]. LCs and dermal DCs are the main cells that induce antigen-specific immunity in the skin, and the two mainstream strategies targeting LCs are either transcutaneous vaccination in situ or manipulating LCs ex vivo to facilitate efficient anti-tumoral response [207]. In the field of transcutaneous cancer vaccines, LCs have emerged as prime targets. Through an endocytic C-type lectin receptor called Langerin (CD207) and a glycomimetic Langerin ligand (liposomes 22), specific and efficient targeting of LCs in human skin has been achieved. These transcutaneous cancer vaccines induce protective T cell immunity, and their effectiveness relies on the efficient activation of LCs [208]. Recently Glyceryl monooleate (MO) was chosen as a skin permeation enhancer, and the MO-based reverse micellar carrier enabled the successful delivery of antigen to Langerhans cells and dermal dendritic cells [209]. (Fig. 5).

## Intestinal resident macrophages

### Homeostasis of tissue resident macrophages in intestine

Intestinal resident macrophages, leveraging their robust phagocytic capacity, play a vital role in maintaining host defense and tissue homeostasis in the gastrointestinal tract [210]. However, unlike other murine tissue-resident macrophages, intestinal macrophages derived from embryos are replaced by circulating monocytes at 3 weeks of age and replenished from peripheral circulating monocytes through a “monocyte waterfall” process depending on CCL2/CCR2 axis [211, 212]. During embryonic development, embryo-derived macrophages turnover, and monocytes defined as Ly6C^hi^CD64^−^CX3CR1^int^MHCII^−^ enter the gastrointestinal tract, transitioning through subsequent differentiation stages to become mature CD64^+^CX3CR1^hi^MHCII^hi^ macrophages in mice [211, 213], and CD64^+^CD11C^+^MHCII^hi^ in human [214]. Among them, the most characterized macrophage population resides in the lamina propria (LP) [210], macrophages in the intestine can be categorized based on their anatomical positioning, primarily distinguishing between lamina propria macrophages (LPMs) and muscularis macrophages (MMs) [13, 215–217]. For many years, it was widely believed that intestinal macrophages were an exception among tissue-resident macrophages, deriving exclusively from circulating monocytes. Recent findings from two research groups have spotlighted a distinct population of long-lived and self-maintain colonic macrophages that endure from early stages into adulthood, without being supplanted by circulating monocytes. These macrophages reside deep within the gut wall, closely associated with blood vessels and enteric neurons of both the submucous and myenteric plexus [13, 82]. This discovery challenges our previous understanding of tissue-resident macrophages in the colon. Recent studies in humans have confirmed the coexistence of long-lived macrophages and those rapidly replaced by incoming monocytes in the intestinal macrophage pool. Notably, the adult small intestine harbors both subsets, with the long-lived macrophages predominantly located in the villi and submucosa [216].

### Characteristics of intestinal resident macrophages

As mentioned above, lamina propria macrophages (LPMs) primarily consist of monocyte-derived macrophages, with a relatively lower proportion of long-lived macrophages in comparison to the deeper intestinal layers. The intestinal lamina propria is characterized by an inflammatory microenvironment resulting from continuous exposure to luminal commensal bacteria and dietary antigens. Despite the presence of this reactive setting, the immune system is tightly controlled to prevent unintentional self-harm, and LPMs play a crucial role as key regulators in establishing a tolerogenic environment [218]. Among the factors involved in immune regulation, IL-10 is the most extensively studied tolerogenic factor in intestinal LPMs, and its complete absence results in the development of spontaneous enterocolitis [219]. In an immature state, LPMs exhibit a failure to upregulate IL-10 and excessive production of inflammatory cytokines such as IL-1β, TNF-α, IL-6, IL-12, and chemokines, that further promotes the influx of LY6C^hi^ monocytes [212, 220]. LPMs also play a crucial role in maintaining the intestinal stem cell niche. Depletion of CSF1R-dependent LPMs impairs paneth cells differentiation and reduces LGR5^+^ stem cells, affecting the differentiation and replenishment of other intestinal epithelial cells [221]. These observations indicate that a subset of CSF1R-dependent LPMs is essential for maintaining intestinal crypt homeostasis. In recent findings, researchers identified that a predominant portion of self-sustaining macrophages in the lamina propria align closely with submucosal neurons and blood vessels [13]. Remarkably, their depletion led to the degeneration of these neurons, vessel disruption, and increased vascular leakage [222].

In contrast to LPMs, a significant portion of intestinal resident macrophages, known as muscularis macrophages (MMs), are derived from embryonic origins and mainly reside in the myenteron, forming a specialized “macrophage niche” within the intestinal environment [13, 82]. Within the myenteric plexus, MMs closely interact with enteric neurons, executing unique functions. Their activation is largely influenced by neuron-derived cues, given their expression of various neurotransmitter receptors that modulate their behavior [215, 223–226]. Moreover, the absence of muscularis macrophages in CSF1op/op mice is associated with a higher density of enteric neurons and a more disorganized structure of the myenteric plexus [217]. Muscularis neuron-associated macrophages within the myenteric plexus play a crucial role in maintaining intestinal homeostasis by synthesizing BMP2, which is essential for orchestrating peristaltic activity [215]. Animal studies have demonstrated that upon pathogen stimulation, MMs upregulate various transcription factors such as NF-Kb, STAT, and P38-MAPK, induce pro-inflammatory gene expression, as well as release chemokines and cytokines including IL-1β, MCP-1, IL-6, and TNF-α [45, 227, 228].

6.3 Intestinal resident macrophages in cancerTissue-resident macrophages in the colon have been identified as TAMs and play a crucial role in the fate of tumors, including their occurrence, development, and elimination [229]. In vitro experiments have demonstrated that TAMs derived from resident macrophages in colorectal cancer exhibit an M2-like phenotype characterized by express high levels of pattern recognition receptors such as MR and CD163, as well as secretion of chemokines including CCL17, CCL22, and CCL24, along with immune regulatory cytokines such as IL-10 and TGF-β. These TAMs play a role in promoting tumor occurrence and development [230]. The transcription factor C-MYC does not involved in macrophage proliferation and survival but is expressed in TAMs and regulates the expression of pro-tumoral genes, such as VEGF, HIF-1α and TGF-β [231]. In addition, it has been demonstrated that lactic acid, a byproduct of tumor cell glycolysis, acts as a potential signal triggering TAMs to adopt an M2 phenotype characterized by the expression of VEGF and Arginase-1, with HIF-1α facilitating this process [232]. In the realm of gastrointestinal neoplasms, tumors of the small intestine, predominantly of the neuroendocrine subtype, manifest infrequently, especially when compared to the higher incidence of colorectal carcinomas [233, 234]. While the direct relationship between these tumors and intestinal macrophages remains underexplored, macrophages play pivotal roles in various inflammatory and tumorigenic processes, suggesting potential involvement in small intestine tumor progression [235]. Consequently, in the subsequent sections, the focus of the review will primarily be on colorectal tumor.

**Fig. 6:**
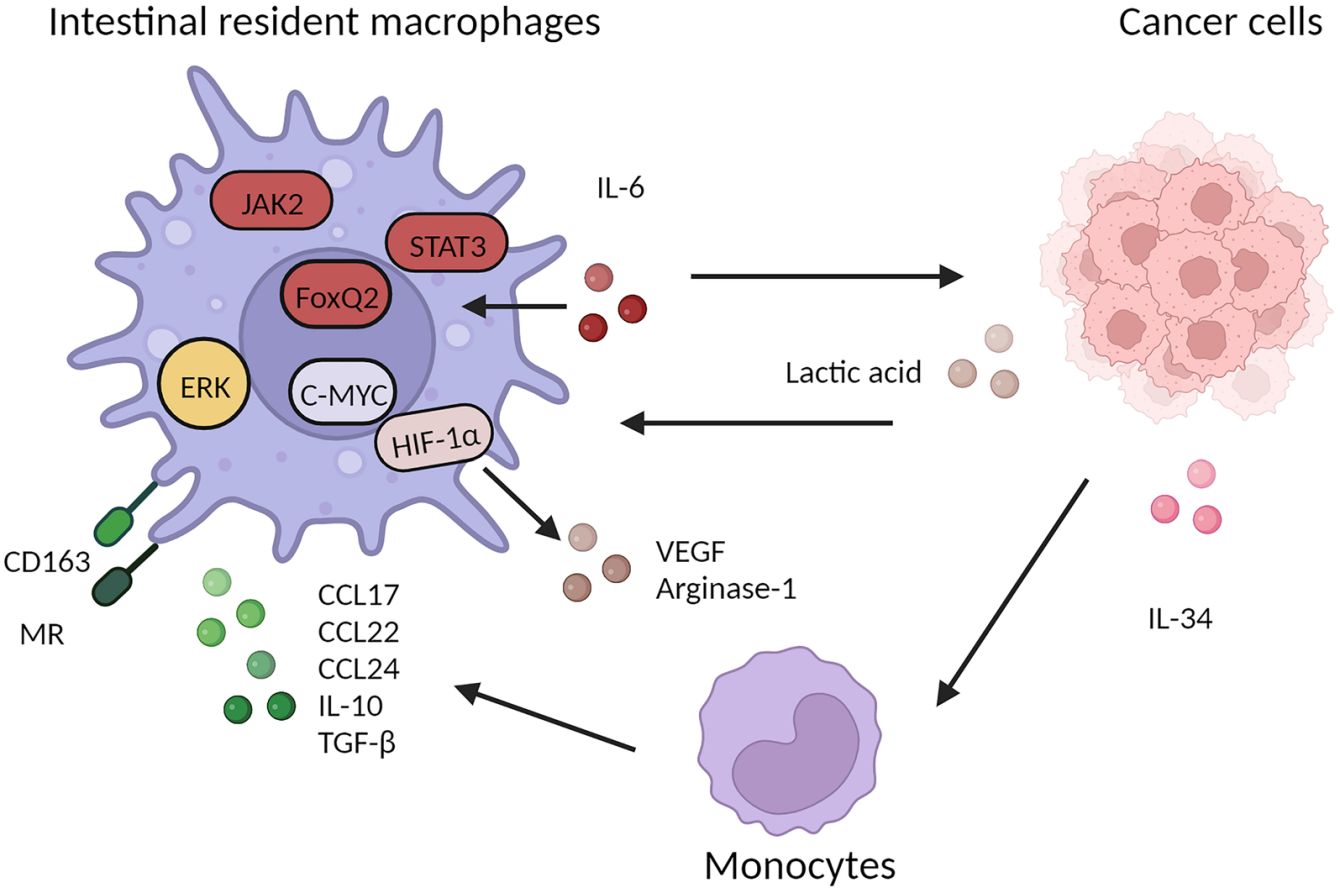
Intestinal resident macrophages facilitate tumor cell invasion. The transcription factor C-MYC regulates the expression of pro-tumoral genes, such as VEGF, HIF-1α and TGF-β in Tumor-associated macrophages (TAMs), which are characterized by the expression of pattern recognition receptors CD163 and MP, as well as the secretion of cytokines IL-10 and TGF-β, as well as chemokines including CCL17, CCL22 and CCL24. Tumor cells produce lactic acid through the mediation of HIF-1α, which induces VEGF and arginase-1 expression in intestinal resident macrophages. Cancer cells secrete IL-34, promoting the polarization of monocyte towards M2 macrophages, thereby further enhancing tumor proliferation and metastasis. TAMs enhance tumor migration and invasion by the ERK signaling pathway, secreting IL-6, and this effect is mediated via the JAK2/STAT3/FoxQ1 axis

Additionally, literature reports have indicated that colon cancer cells secrete IL-34, influencing the polarization of monocytes in the mucosal lamina propria towards M2 macrophages expressing CD206 and CD163, as well as secreting IL-10., thereby further enhancing tumor proliferation and metastasis [236]. Similarly, Irene Soncin et al. found a resident subsets CCR2^−^independent F4/80^+^MHCII^lo^ macrophage in the microenvironment of colorectal adenoma, which had self-renewal capacity in TME and played pro-tumorigenic roles in tumor progression. However, the process of self-renewal depended on isolated niche and CSF1 may be a key facilitator [237]. In addition, a portion of CD169^+^ macrophages (characterized by CD115^+^CD169^+^CD11B^+^F4/80^lo^CD11C^lo^), reside in the colonic lamina propria, primarily surround the crypt and arise from both tissue macrophage self-renewal and blood stem cell input, with their development relying on vitamin A [238]. The presence of these cells in adjacent lymph nodes of colon cancer patients correlates with clinical stage, overall survival, and prognosis [239], with a higher concentration suggesting extended survival and a positive clinical outlook for individuals with tumors [239]. Colonic macrophages also play a significant role in metastatic tumors. Lim, S.Y. et al. elucidated that TAMs prompt the production of S100A8/A9 mRNA within the colon cancer TME through the ERK signaling pathway, subsequently enhancing tumor migration [240]. On a related note, Wei, C. et al. highlighted that TAMs, by secreting IL-6, instigate the EMT process in colorectal cancer, bolstering its migration and invasion via the JAK2/STAT3/ FOXQ1 axis [241]. Both intestinal resident macrophages replenished from peripheral circulating monocytes and the newly discovered embryos-derived and self-renewal macrophages have shown the characteristics of promoting tumors, although they show different functions in maintaining tissue homeostasis. Therefore, the concept of “intestinal resident macrophage” is still attractive candidates for therapeutic intervention. However, it is still unclear of the tumor promoting capacity of these two populations of resident macrophages. This also presents a major challenge of the potential therapeutic targets on exhausted macrophages and reprogram macrophage phenotypes [242, 243]. It is well believed that an in-depth understanding of its function and underlying mechanism will help us develop effective macrophage-based therapeutic approaches (Fig. 6).

## Conclusions

The advances and progress of fate-mapping, parabiosis models and single cell sequencing techniques facilitates the better understanding of macrophage function regarding their origin during past decade [67]. Although the maintain and expansion of TRMs are instructed by three main factors derived from stromal cells (CSF1, CSF2 and IL-34) via two receptors on macrophages (CSF1R and CSF2R) [244], niche specific signals also played central roles in governing TRMs such as TGF-β signaling controlling PPAR-γ in alveolar macrophages [41], and retinoic acid signaling regulating GATA6 expression in peritoneal cavity-resident macrophages [245]. The roles of TRMs in regulating cancer growth is organ specific in that the expansion of TRMs and flooding influx of bone marrow derived macrophage varied among organs [3]. The number of TRMs decreased while BM-derived monocytes expanded in murine breast cancer model [246, 247]. While in a pancreatic cancer model, TRMs gradually increased [248]. As a result, depletion of bone marrow derived circulation monocyte leads to tumor shrinkage in breast cancer model and ablation of TRMs significantly impaired tumor growth in pancreatic adenocarcinoma model [6, 246–248]. Apart from varied roles of TRMs in primary cancer, their functions in regulating metastatic cancer could be quite different. In a KCs-deficient mice, the number of colorectal carcinoma metastasis increased [249]. While depletion of alveolar macrophages reduced metastatic tumor spreading [250]. Additionally, in breast cancer primary tumors, FOLR2^+^ tissue-resident macrophages are positioned in the perivascular areas of the tumor stroma, where they interact with CD8^+^ T cells, and their density in tumors is positively associated with improved patient survival rates [251]. TIM-4^+^ cavity-resident macrophages have been associated with a reduction in the population of CD8^+^ T cells. Mechanistically, CD8^+^ T cells upregulate phosphatidylserine, rendering them susceptible to being sequestered away from tumor targets and suppressed by the proliferating TIM-4^+^ cavity-resident macrophages. However, blocking TIM-4 effectively prevents this sequestration and proliferation-induced suppression, leading to enhance effectiveness of anti-PD-1 therapy and adoptive T cell therapy in mouse models by enabling improved tumor targeting and activation of CD8^+^ T cells [252]. Specific depletion of CD163^+^ TIM-4^+^ macrophages prevent metastasis of ovarian cancer [253, 254]. The distinct macrophage lineage may constantly adjust themselves to environmental cues, adding the complexity of phenotypic study. Ultimately, the dynamics of TRMs and BM-derived monocytes at different stages of cancer progression and niche specific markers as well as the microenvironment profiles should all be investigated for a better understanding before tissue specific macrophages can be therapeutically targeted.

## Data Availability

Not applicable.

## Abbreviations

TRMs: Tissue resident macrophages
TAMs: Tumor associated macrophages
BMDMs: Bone marrow derived macrophages
BM: Bone marrow
GM-CSF: Granulocyte–macrophage colony-stimulating factor
LYVE: Lymphatic vessel endothelial hyaluronan receptor
MHCII: Major histocompatibility complex class II
AMs: Alveolar macrophages
NAMs: Nerve-associated interstitial macrophages
IMs: Interstitial macrophages
TGF-βR: Transforming growth factor-beta receptor
PPAR-γ: Peroxisome proliferator-activated receptor gamma
CSF1R: Colony-stimulating factor 1-receptor
HLA-DR: Human leukocyte antigen-DR
MERTK: Mer tyrosine kinase
SIGLECF: Sialic acid-binding immunoglobulin-like lectin F
FABP4: Fatty acid-binding protein 4
MARCO: Macrophage receptor with collagenous structure
MRC1: Mannose receptor c-type 1
MSR1: Macrophage scavenger receptor 1
TGF-β: Transforming growth factor beta
IL-10: Interleukin-10
IL-12: Interleukin-12
IL-4: Interleukin-4
IL-6: Interleukin-6
IL-8: Interleukin-8
IL-1β: Interleukin-1 beta
PGE2: Prostaglandin E2
INOS: Inducible nitric oxide synthase
TNF-α: Tumor necrosis factor alpha
MIP1α: Macrophage inflammatory protein-1 alpha
CX3CR1: C-X3-C motif chemokine receptor 1
CCR2: C–C chemokine receptor type2
TGF-β2: Transforming Growth Factor-beta 2
PLAUR: Plasminogen Activator Urokinase Receptor
FCNA: Fibronectin 1
NSCLC: Non-small cell lung cancer
CXCR1: C-X-C chemokine receptor 1
CTL: Cytotoxic T cell
ROS: Reactive oxygen species
BACH1: BTB and cnc homology 1
PDLIM2: PDZ and lim domain 2
STAT3: Signal transducer and activator of transcription 3
SOCS1: Suppressor of Cytokine Signaling 1
INHBA: Inhibin beta A
SOCS3: Suppressor of cytokine signaling 3
EMT: Epithelial-mesenchymal transition
IL-12β: Interleukin-12 beta
IL-1α: Interleukin-1 alpha
MO-MDSC: Monocytic-myeloid-derived suppressor cells
CXCL10: C-X-C motif chemokine ligand 10
CXCR3: C-X-C chemokine receptor 3
TLR4: Toll-like receptor 4
CCL12: C–C motif chemokine ligand 12
WNT: Wingless-related integration site
KCs: Kupffer cells
TGF-β1: Transforming growth factor beta 1
CXCL1: C-X-C motif chemokine ligand 1
CXCL2: C-X-C motif chemokine ligand 2
CXCL8: C-X-C motif chemokine ligand 8
CXCL12: C-X-C motif chemokine ligand 12
CXCL16: C-X-C motif chemokine ligand 16
CCL2: C–C motif chemokine ligand 2
YAP: Yes-associated protein
M-CSF: Macrophage-colony stimulating factor
IL-34: Interleukin-34
ID3: Inhibitor of DNA binding 3
LXR-α: Liver X receptor-alpha
ZEB2: Zinc finger e-box binding homeobox 2
DLL4: Delta-like ligand 4
TIM-4: T-cell immunoglobulin and mucin domain-containing protein 4
CLEC4F: C-type lectin domain family 4 member F
TLRs: Toll-like receptors
TLR4: Toll-like receptor 4
TLR9: Toll-like receptor 9
PD-L1: Programmed cell death-ligand 1
PD-1: Programmed death 1
TIM-3: T cell immunoglobulin domain and mucin domain-3
ESAM: Endothelial cell-selective adhesion molecule
HCC: Hepatocellular carcinoma
APCs: Antigen presenting cells
FOLR2: Folate receptor beta
MT1G: Metallothionein 1G
TIGIT: T cell immunoreceptor with Ig and ITIM domains
CTLA4: Cytotoxic T-lymphocyte-associated protein 4
TREM-1: Triggering receptor expressed on myeloid cells 1
TNF: Tumor necrosis factor
VEGF: Vascular endothelial growth factor
COX-2: Cyclooxygenase-2
HGF: Hepatocyte growth factor
MMP-9: Matrix metalloproteinase-9
LCs: Langerhans cells
DMs: Dermal macrophages
DCs: Dendritic cells
PU.1: Spi-1 proto-oncogene transcription factor
BMPR1A: Bone morphogenetic protein receptor type 1a
ALK3: Activin receptor-like kinase 3
RUNX3: Runt-related transcription factor
ID2: Inhibitor of DNA binding 2
Smad2: Mothers against decapentaplegic homolog 2
Smad3: Mothers against decapentaplegic homolog 3
Smad4: Mothers against decapentaplegic homolog 4
Smad7: Mothers against decapentaplegic homolog 7
MAFB: V-MAF avian musculoaponeurotic fibrosarcoma oncogene homolog B
C-maf: Cellular-musculoaponeurotic fibrosarcoma oncogene homolog
ZBTB46: Zinc finger and BTB domain-containing protein 46
CD207: C-type lectin receptor langerin
EPCAM: Epithelial cell adhesion molecule
CCR7: C–C chemokine receptor 7
CXCR4: C-X-C chemokine receptor 4
EGR1: Early growth response 1
SCC: Squamous cell carcinoma
BCC: Basal cell carcinoma
IFN-γ: Interferon-gamma
PAK1: P21-activated kinase 1
CNS: Central nervous system
IGF-1: Insulin-like growth factor-1
SALL1: Spalt-like transcription factor 1
IRF-8: Interferon-regulatory factor 8
TMEM119: Transmembrane protein 119
P2RY12: Purinergic receptor P2Y g-protein coupled 12
CXCR3: C-X-C chemokine receptor 3
IBA-1: Ionized calcium-binding adapter molecule 1
MYB: Myeloblastosis oncogene
EGF: Epidermal growth factor
STI1: Stress-inducible protein 1
CCR2: C–C chemokine receptor 2
NSCLC-BM: Non-small cell lung cancer develop brain metastasis
JAK2: Janus kinase 2
STAT3: Signal transducer and activator of transcription 3
ANXA1: Annexin A1
CpG-C: CPG oligodeoxynucleotide type c
SIRPα: Signal regulatory protein alpha
TME: Tumor microenvironment
LPS: Lipopolysaccharide
MMTV-Wnt1: Mouse mammary tumor virus- wingless-type MMTV integration site family, member 1
LP: Lamina propria
LPMs: Lamina propria macrophages
MMs: Muscularis macrophages
MO: Monooleate
LGR5: Leucine-rich repeat-containing g-protein coupled receptor 5
BMP2: Bone morphogenetic protein 2
NF-KB: Nuclear factor kappa B
STAT: Signal transducer and activator of transcription, Activator of transcription
P38-MAPK: P38 mitogen-activated protein kinase
MCP-1: Monocyte chemoattractant protein-1
MR: Mannose receptor
CCL17: C–C motif chemokine ligand 17
CCL22: C–C motif chemokine ligand 22
CCL24: C–C motif chemokine ligand 24
C-MYC: Cellular myelocytomatosis oncogene
HIF-1α: Hypoxia-inducible factor-1 alpha
ERK: Extracellular signal-regulated kinase
MET: Epithelial-Mesenchymal Transition
FOXQ1: Forkhead box q1
CSF2: Colony-stimulating factor 2
CSF2R: Colony-stimulating factor 2-receptor
GATA6: GATA-binding protein 6
FOLR2: Folate Receptor 2
